# Comparison of the Proteomes of Porcine Macrophages and a Stable Porcine Cell Line after Infection with African Swine Fever Virus

**DOI:** 10.3390/v13112198

**Published:** 2021-11-01

**Authors:** Elisabeth Wöhnke, Walter Fuchs, Luise Hartmann, Ulrike Blohm, Sandra Blome, Thomas C. Mettenleiter, Axel Karger

**Affiliations:** 1 Institute of Molecular Virology and Cell Biology, Friedrich-Loeffler-Institut, Federal Research Institute for Animal Health, Südufer 10, 17493 Greifswald, Germany; elisabeth.woehnke@fli.de (E.W.); walter.fuchs@fli.de (W.F.); 2 Institute of Immunology, Friedrich-Loeffler-Institut, Federal Research Institute for Animal Health, Südufer 10, 17493 Greifswald, Germany; luise.hartmann@fli.de (L.H.); ulrike.blohm@fli.de (U.B.); 3 Institute of Diagnostic Virology, Friedrich-Loeffler-Institut, Federal Research Institute for Animal Health, Südufer 10, 17493 Greifswald, Germany; sandra.blome@fli.de; 4 Friedrich-Loeffler-Institut, Federal Research Institute for Animal Health, Südufer 10, 17493 Greifswald, Germany; thomas.mettenleiter@fli.de

**Keywords:** African swine fever virus, proteome, primary cell culture, mass spectrometry

## Abstract

African swine fever virus (ASFV), causing an OIE-notifiable viral disease of swine, is spreading over the Eurasian continent and threatening the global pig industry. Here, we conducted the first proteome analysis of ASFV-infected primary porcine monocyte-derived macrophages (moMΦ). In parallel to moMΦ isolated from different pigs, the stable porcine cell line WSL-R was infected with a recombinant of ASFV genotype IX strain “Kenya1033”. The outcome of the infections was compared via quantitative mass spectrometry (MS)-based proteome analysis. Major differences with respect to the expression of viral proteins or the host cell response were not observed. However, cell-specific expression of some individual viral proteins did occur. The observed modulations of the host proteome were mainly related to cell characteristics and function. Overall, we conclude that both infection models are suitable for use in the study of ASFV infection in vitro.

## 1. Introduction

African swine fever virus (ASFV, family *Asfarviridae*) is a highly virulent virus that infects swine (family *Suidae*) for which, currently, neither a vaccine nor a treatment is available. Its double-stranded DNA-genome contains more than 150 open reading frames (ORFs). However, for many of them, information regarding functions and interactions is still limited. For roughly one third of the ASFV ORFeome, no information about the expression of a corresponding protein in mammalian cells is available [1]. This knowledge gap, which is partially due to the lack of appropriate immunologic reagents, has been partially filled by the application of MS-based proteomics using infected stable cell cultures [2,3] but expression data are still missing for ASFV-infected macrophages, the natural target cell of ASFV. The genome of ASFV isolates is quite variable. While homologs of a core set of conserved viral genes encoding for viral structural proteins or proteins involved in virion morphogenesis can be found in all 24 genotypes currently defined, the composition of a genome can vary with respect to the presence or absence of other genes [4]; among them are sets of paralogous genes known as multi gene families (MGFs). MGF proteins have been described as virulence factors, host range determinants or modulators of host response to infection [5,6,7,8]. Like other viruses, ASFV is known to evade the antiviral response by modulating immune-related pathways, such as NFκB-mediated signaling or the interferon response. The viral proteins directly involved in immune evasion are only known for some of these pathways (reviewed in [9]).

Macrophages, the primary target cells of ASFV [10], are phagocytic cells belonging to the myeloid lineage that differentiate from monocytes in response to growth factors such as granulocyte-monocyte colony-stimulating factor (GM-CSF) [11,12]. As professional antigen-presenting cells, macrophages express swine leukocyte antigens (SLAs) I and II, homologues of human major histocompatibility complexes I and II, on their surface. This complex is formed by SLA-DR and SLA-DQ proteins, both consisting of alpha and beta chains encoded by SLA-DRA and SLA-DRB or by SLA-DQ1 and SLA-DQB genes, respectively [13]. Apart from SLA complexes, swine macrophages are characterized by the expression of specific clusters of differentiation (CD) proteins such as CD14, CD172a (SWC3), CD68 and the scavenger receptor CD163 [11,14].

Many studies about ASFV replication and host interactions have been performed using established cell culture models, such as Vero cells infected with cell culture adapted viruses such as BA71V [15]. In this way, some pitfalls that come with the use of primary cell cultures in infection experiments (animal health issues, higher variability) can be avoided, but presumably at the cost of a more artificial infection model.

While the transcriptomes of ASFV-infected cell lines [16] or primary macrophages have been studied in vitro [16,17] and in vivo [18], proteome studies of ASFV-infected cells [2] or ASFV particles [3,19] using shotgun MS platforms are rare [20]. A recent proteome study with ASFV-infected porcine alveolar macrophages (PAMs) noted a differential regulation of host proteins involved in immune processes and defense responses [21]. Older studies of the ASFV proteome using 2D electrophoresis, optionally combined with MALDI-TOF MS, identified a limited number of proteins that were influenced by the infection, among them heat shock proteins, redox-related proteins, and nucleoside diphosphate kinases [22,23,24,25,26,27,28].

Here, we conducted the first MS-based quantitative proteome analysis of in vitro ASFV-infected primary porcine monocyte-derived macrophages. For comparison, the stable wild-boar-derived cell line WSL-R [29], which is commonly used for in vitro infection experiments with ASFV, was analyzed in parallel to monitor any differences in the expression patterns of ASFV proteins in primary cells and a stable cell line. The modulation of the host proteome after infection was also monitored via MS analysis and compared between the two cell types using biostatistical means. ASFV-Kenya1033 was isolated from domestic pigs in western Kenya as a virulent genotype IX strain (L. Steinaa & R. Bishop, personal communication). For this study, the recombinant ASFV-Kenya1033-ΔCD2v-dsRed was chosen for its proven ability to replicate well in both WSL-R cells [30] and macrophages [31], which is prerequisite for a proteome analysis focusing on the cell-specific expression of viral and host genes after infection with ASFV.

## 2. Materials and Methods

### 2.1. Safety Statement

All ASFV-infection experiments were carried out in a biocontainment facility fulfilling the safety requirements for ASF laboratories and animal facilities (Commission Decision 2003/422/EC, Chapter VIII).

### 2.2. Cells and Viruses

The wild boar lung-derived cell line WSL-R (hereafter ‘WSL’ cells) [29], obtained from the Biobank of the FLI (CCLV-RIE #0379), was cultured in Iscove′s modified Dulbecco’s medium mixed with Ham’s F-12 nutrient mix (1:1; *v*/*v*) supplemented with 10% fetal bovine serum (FBS) (during cultivation) or 5% FBS (after inoculation).

Virus stock of ASFV-Kenya1033-ΔCD2v-dsRed [30] was grown on WSL cells. Titers were determined on WSL cells and peripheral blood monocytic cells (PBMCs) as TCID_50_/mL [32]. ASFV-Kenya1033-ΔCD2v-dsRed, derived from highly pathogenic genotype IX isolate ASFV-Kenya1033 (isolated February 2013), expressed dsRed as a fluorescent reporter under the late p72 promotor in the CD2v locus. The generation and partial characterization of the recombinant has been described elsewhere [30]. Both viruses replicate well in WSL cells, but titers of the recombinant are slightly reduced in comparison to the parental virus.

### 2.3. Isolation and Cultivation of Porcine Macrophages

Blood was drawn from 6- to 12-month-old female domestic pigs kept at the FLI animal facility (permission: LALLF-Nr. 7221.3-2-041/17). PBMCs were obtained using Pancoll animal density gradient medium (PanBiotech, Aidenbach, Germany) with a density of 1.077 g/mL, in accordance with the manufacturer’s recommendations. For the magnetic cell separation of CD172a^+^ PBMCs, anti-mouse IgG1 magnetic particles (BD Biosciences, Franklin Lakes, NJ, USA) in combination with α-SWC3 hybridome supernatant (clone 74-22-15) were used according to established procedures. CD172a^+^ PBMCs were cultured in Primaria^TM^ cell culture dishes (Corning, New York, NY, USA) with PC-1^TM^ Medium (Lonza, Basel, Switzerland) containing 1% penicillin/streptomycin solution (*v*/*v*, ThermoFisher Scientific, Waltham, MN, USA). Cells were incubated at 37 °C and 2.5% CO_2_.

Differentiation into mature moMΦ was induced using a modified protocol based on Carlson et al., 2016 [33] and Carlson et al., 2020 [31]. Briefly, one day after isolation, cells were washed three times with PBS before treatment with 5 ng/mL GM-CSF (KingFisher Biotech, Saint Paul, USA, #RP0940S) applied in fresh culture medium.

### 2.4. Phenotypic Characterization of Monocyte Derived Macrophages

For fluorescence analysis, moMΦ were grown on glass cover slides and infected with ASFV-Kenya1033-ΔCD2v-dsRed 24 h post differentiation using a MOI of 2. At 6 or 18 hpi, moMΦ were fixed in 3.75% (*v*/*v*) formaldehyde in PBS, permeabilized with 0.5% Triton X-100 in PBS and stained for actin using an Alexa568-conjugated phalloidin derivate (0.33 µM, Invitrogen). Viral proteins p30 and p72 were detected using monospecific rabbit antisera [30,34] and AlexaFluor 647 conjugated secondary antibodies. Nuclei were stained using Hoechst33342 (Invitrogen, Waltham, MA, USA). Samples were analyzed using a Leica DMi8 microscope. Pictures were processed using ImageJ (v1.52 h, U. S. National Institutes of Health, Bethesda, MD, USA) software.

To assess the expression of CD14, CD172a, CD163, SLA II and CD68 in moMΦ via flow cytometry, the cells were harvested 24 h after differentiation, washed in FACS buffer (0.1% sodium azide, 0.1% FCS, 1 mM EDTA in PBS), sedimented (350× *g*, 4 °C, 5 min) and stained for surface epitopes CD14, CD172a, CD163 and SLAII (10 min, 4 °C, dark) using appropriate antibodies; for details, see Table S1. Cell viability was checked with Zombie Aqua staining reagent (Biolegend, San Diego, CA, USA) in accordance with the manufacturer’s directions. After fixation and permeabilization with True Nuclear Transcription Factor Buffer Set (Biolegend), intracellular CD68 was stained for 15 min at room temperature (RT) in the dark. Samples were measured with a BD FACSCanto™ II (BD Biosciences, BD FACSDiva™ Software v9.0.1, BD Biosciences, Franklin Lakes, NJ, USA) flow cytometer and the results processed via FlowJo™ v10.7 (BD Biosciences, Franklin Lakes, NJ, USA) software. See Figure S1 for details of the gating strategy.

### 2.5. Infection with ASFV-Kenya1033-ΔCD2v-dsRed

To enhance infection rates, cells were centrifuged (60 min, 37 °C, 600× *g*) during inoculation with ASFV-Kenya1033-ΔCD2v-dsRed with MOI2 ([30], JH Forth, L Käbisch, R Portugal, S Blome, GM Keil, manuscript in preparation).

For proteome analysis of moMΦ, approximately 5 × 10^6^ cells were detached from the cell culture plates 24 hpi via incubation with 4 °C cold PBS supplemented with 50 mM EDTA, pelleted (250× *g*, 5 min, 4 °C) and harvested in 250 µL of lysis buffer (2% SDS in 100 mM Tris-HCl, pH 8.0). WSL cells were washed with PBS three times and harvested 48 hpi in 500 µL of lysis buffer. The longer incubation time for WSL cells was chosen to ensure that all cells were infected and had progressed to the late stage of infection [2,30]. For lysis and the inactivation of ASFV, the mixtures were incubated at 95 °C for 10 min, cooled to RT and clarified by centrifugation (5 min, 10,000× *g*, 20 °C). This supernatant is referred to as ‘lysate’.

### 2.6. Generation and Analysis of MS-Samples

For shot-gun proteomic analysis, lysates were supplemented with 0.5% dithiothreitol for reduction and digested by Filter Aided Sample Preparation (FASP) [35] using Trypsin (Promega #V5111, Madison, WI, USA) at an enzyme to substrate ratio of 1:50. Desalted peptides were separated using nano reversed phase liquid chromatography. Per sample, 1360 fractions were spotted to a MALDI target plate and analyzed via MALDI-TOF/TOF MS with an Ultraflextreme instrument (Bruker, Bremen, Germany). For details see Supplementary Methods.

Protein abundances in mol percent (%mol) were calculated on the basis of the exponentially modified protein abundance index (emPAI) [36]. MS data are available at JPOST under the identifier JPST001339 (https://repository.jpostdb.org/entry/JPST001339).

### 2.7. Statistics and Software

All calculations were performed using the statistical language R [37]; graphs were generated with the R-package ggplot2 [38] and with BioRender.com (accessed on 30 September 2021).

The following study design was applied: moMΦ were isolated from three pigs. From one animal, mock-infected and ASFV-infected cells were analyzed in triplicate together with mock and infected triplicates of WSL cells. Infected moMΦ from the other two animals were also analyzed in triplicate to assess the variability of ASFV protein expression in cells from different animals. For the biostatistical analysis of host protein expression levels, porcine protein identifiers were referenced to the corresponding genes using the R-package gprofiler2 (version 0.2.1) [39]. Gene products were only considered for statistical analysis if they were expressed in at least one of the three replicates from two of the three pigs or in two of the three WSL cell samples. Genes were only considered as differentially expressed (differentially expressed genes, DEG) under different conditions if expression levels changed more than 2-fold and *p*-values of a two-sided *t*-test were <0.05.

Host gene lists for enrichment analysis of Gene Ontology (GO) terms [40] or Kyoto Encyclopedia of Genes and Genomes (KEGG) pathways [41] were compiled from the output lists of ProteinScape software using R scripts. These lists contained DEGs and also the genes that were identified only under one of the compared conditions (infected versus naïve or moMΦ versus WSL). GO term and KEGG pathway enrichment analysis was performed using CytoScape (version 3.8.2) [42] together with the package ClueGO (version v2.2.5.7) [43].

## 3. Results

**Figure 1:**
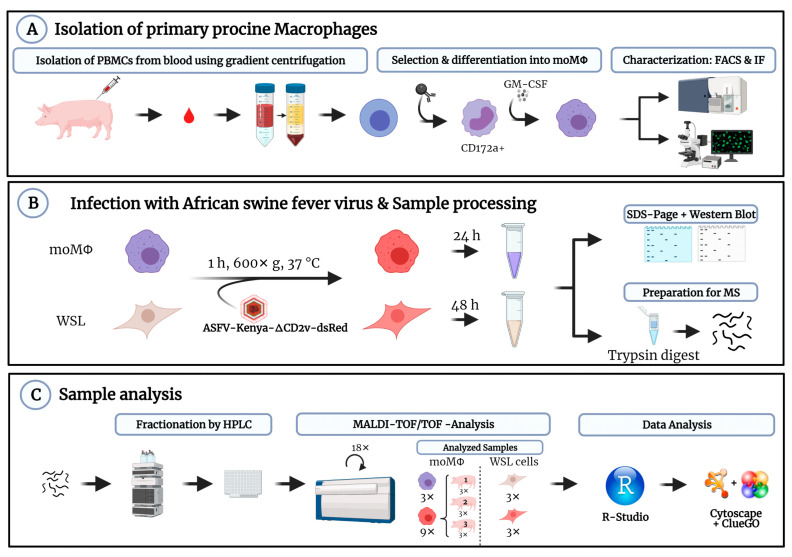
Workflow for generation, processing and analysis of samples for proteome analysis. (**A**) Isolation, selection and differentiation one day after isolation and quality control of primary moMΦ, (**B**) ASFV infection, sample generation and processing for MS analysis, (**C**) analysis of peptides by HPLC-MALDI-TOF/TOF MS workflow and data processing.

The goal of this study was to compare the expression of ASFV genes and host response in primary porcine moMΦ and WSL cells. Briefly, moMΦ were differentiated from PBMC-derived, magnetically sorted CD172a^+^ monocytes, characterized and infected with ASFV. Proteomes of ASFV-infected moMΦ and WSL cells were compared via HPLC-MS analysis following the workflow outlined in Figure 1.

In order to achieve the high infection rates required for the unbiased comparison of both cell types, moMΦ and WSL cells were harvested at 24 hpi and 48 hpi [2], respectively.

### 3.1. Characterization of Primary Macrophages

**Figure 2:**
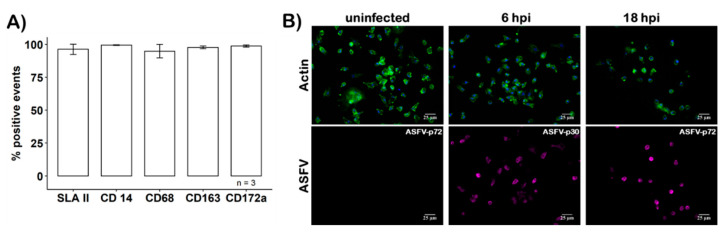
Characterization of moMΦ. (**A**) Phenotypic characterization of moMΦ by flow cytometry. Surface markers SLA class II, CD14, CD68, CD163 and CD172a are expressed on >95% of all moMΦ. (**B**) Morphologic characterization by fluorescence microscopy. Representative data from different preparations are shown. moMΦ were infected with ASFV at MOI2, fixed at 6 or 18 hpi, and stained for actin with phalloidin (green) to visualize the cell cortex. Early (p30, 6 hpi) or late (p72, 18 hpi) ASFV proteins were immunostained (violet) with appropriate antibodies. Bar indicates 25 µm.

**Figure 3:**
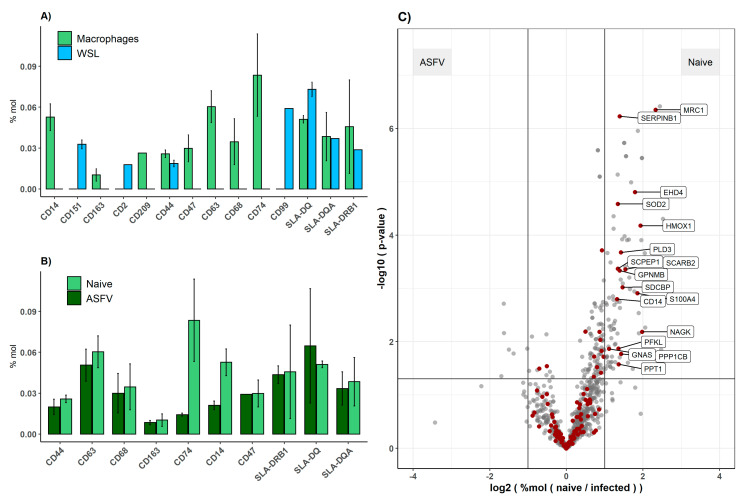
Comparison of host protein expression between WSL and moMΦ and in response to ASFV infection. Expression of CD markers and SLA II in naïve WSL and moMΦ (**A**) and infected and mock-infected moMΦ (**B**). Volcano plots representing host gene expression levels in response to infection in moMΦ (**C**). The horizontal line corresponds to *p* = 0.05 (two-sided *t*-test), vertical lines to fold-changes of 2 and 0.5, respectively, red dots indicate host genes highlighted in previous ASFV-related publications.

To avoid the accumulation of BSA from the medium by the moMΦ, BSA- and serum-free culture conditions had to be used. Under these conditions, the prepared cells expressed a panel of moMΦ-specific CD markers which were assessed via flow cytometry (Figure 2A), maintained their characteristic morphology, and remained susceptible to ASFV infection, as confirmed using immunofluorescence microscopy (Figure 2B). Additionally, macrophage characteristic CD markers were detected by MS in moMΦ but not in WSL cells (Figure 3A).

To confirm the moMΦ phenotype using protein expression profiling, we performed GO term enrichment and GO term clustering analysis of genes that were differentially expressed in naïve moMΦ and WSL cells using CytoScape and ClueGO software (Supplementary file ‘S5_Gene_lists.xlsx’ sheet ‘Gene lists for EA’). Typical biological pathways related to phagocytic antigen-presenting cell functions such as endocytosis (GO:0006897; KEGG:04144), phagocytosis (GO:0006909; KEGG:04145), signal transduction (GO:0007165; GO:0007186) and immune-related pathways (KEGG:04612) were over-represented (Bonferroni-corrected *p*-value < 0.05) in moMΦ (Supplementary Table S5 ‘S5_Gene_lists.xlsx’, sheet ‘Results enrichment analysis’). DEGs overexpressed in moMΦ included the macrophage mannose receptor (MRC1), the superoxide dismutase (SOD2), biliverdin reductase (BLVRB) and 11 genes related to the lysosome (KEGG:04142), such as Cathepsin A (CTSA) and ß-hexosaminidase subunits α and β (HEXA, HEXB). Vice versa, some proteins linked to the fatty acid metabolism (FASN, FABP3) or the TNF receptor-associated protein 1 (TRAP1) were overexpressed in WSL (Supplementary File ‘S4_MS-statistics.xlsx’, sheet ‘Q_Cell type’, Figure S4a).

These results indicated that the typical characteristics of protein expression in moMΦ had been maintained during cultivation and that the proteome analysis reflected cell-type-specific characteristics.

### 3.2. Infection of moMΦ by ASFV

As animal-to-animal variation is a major general caveat of experiments using primary cells, the reproducibility of host and viral protein expression after the infection of moMΦ was of prime importance. To test this, moMΦ from three different animals were cultured and infected, and the proteomes were analyzed 24 hpi. Prior to MS analysis, ASFV infection was confirmed via the expression of the dsRed reporter and immunoblot analysis. Additionally, sample homogeneity was verified via SDS PAGE of the proteins followed by Coomassie staining (Figure S2).

**Figure 4:**
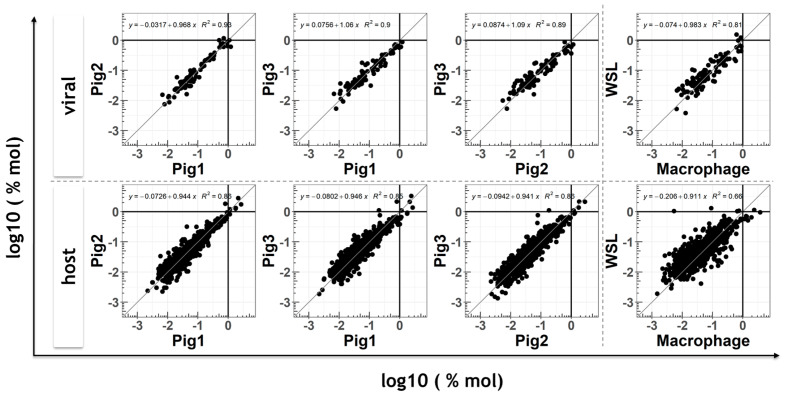
Comparison of viral and host gene expression levels. Scatter plots of viral (upper row) and host (lower row) protein levels representing moMΦ preparations from different pigs and WSL cells (far right panels). Mean expression levels (%mol concentrations) of at least 3 biological replicates are shown after log10 transformation (data taken from Supplementary Table ‘S7_MS-quantification.xlsx’). N = 3 for the comparison of individual pigs. In the right panels, the means of 9 moMΦ and 3 WSL samples are shown. The dissecting lines are shown as references in gray.

**Figure 5:**
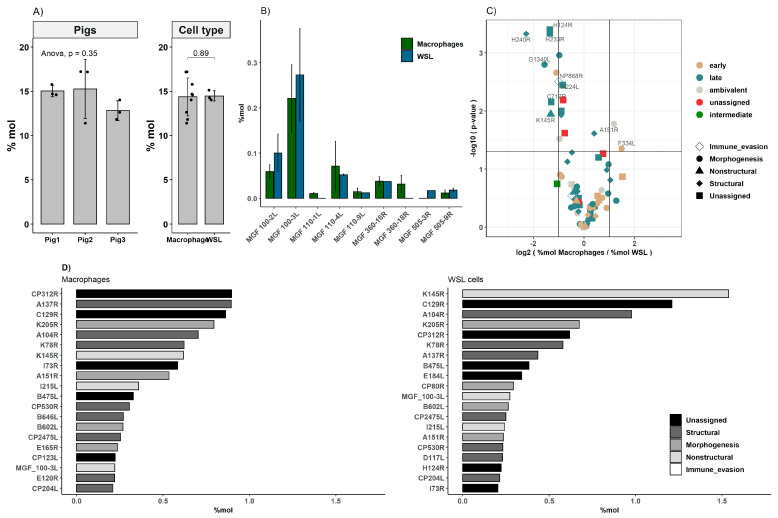
Expression of viral proteins in moMΦ and WSL cells. (**A**) Quantitative analysis of viral protein expression as proportion of whole proteome. (**B**) Expression of MGF members. (**C**) Volcano plot representing differential expression of proteins belonging to different functional group and kinetic classes, the horizontal line corresponds to *p* = 0.05 (two-sided *t*-test), vertical lines to fold-changes of 2 and 0.5, respectively, n(moMΦ) = 9, n(WSL) = 3. (**D**) Mean expression levels in mole percent (%mol) of most abundantly expressed viral proteins in moMΦ and WSL cells.

Protein expression levels were calculated for all three experiments and are compared in the scatterplots shown in Figure 4. Although some degree of variation was observed, the mean expression levels of ASFV and host proteins correlated well between different animals with correlation coefficients ranging from 0.89 to 0.93 for viral proteins and 0.85 to 0.86 for host proteins. Together with the observed mean relative standard deviations of protein expression levels (viral proteins: 0.36 to 0.45; host proteins: 0.32 to 0.36) (Table S2), the reproducibility of viral expression levels (Figure S3) of biological replicates from different pigs, the good accordance of the proportion of viral proteins in relation to the whole cell proteome (Figure 5A), and the high infection rates (Figure S2a) indicated that moMΦ could be experimentally infected in a reproducible way. 

### 3.3. Comparison of Viral Proteomes Expressed in moMΦ and WSL Cells

As expected, a higher degree of variation was noted when the expression levels of individual proteins were compared between the different cell types, moMΦ and WSL cells (Figure 4, far right panels). Here, the correlation coefficients were 0.81 and 0.66 for viral and host proteins, respectively. As expected, the mean relative standard deviations of the expression levels of individual host proteins were lower in WSL cells (0.28 for naïve and 0.31 for ASFV-infected cells) than in moMΦ (0.30 for naïve and 0.36 for ASFV-infected cells) (Table S2), indicating that animal-to-animal variation did contribute to the variation of calculated protein expression levels more than the batch-to-batch variation of WSL cells.

In total, 115 viral proteins, representing 112 ASFV-ORFs, were detected with an overlap of 101 present in both cell types. While ten proteins were only detected in infected moMΦ, four were exclusively found in WSL cells. One of the proteins exclusively identified in moMΦ was p37, a cleavage product of pp220 (ORF CP2475L). Additionally, the presence of N-terminal peptides of p150 and p8, derived from pp220 and pp60, respectively, confirmed the proteolytic processing of polyproteins (Figure S5, Supplementary File ‘S4_MS-statistics.xlsx’, sheet ‘MS Data Viral Proteins’).

The corresponding proteins of eleven viral ORFs were identified, of which so far, only transcripts have been reported; among them were pK196R, pE301R and pB385R. In total, nine MGF members were detected; for five of these, a corresponding protein was detected for the first time. Six members of MGFs were present in both cell types. MGF 505-3R could only be identified in WSL, while MGF 360-18R and MGF 110-1L were only present in moMΦ (Figure 5B).

The levels of viral proteins that were expressed in both WSL cells and moMΦ were analyzed in detail (Figure 5, Table 1). Together, ASFV proteins accounted for roughly 14–15% of the total protein content in both moMΦ and in WSL cells, with some variation between the different primary cell preparations. We observed no biases in the expression levels of viral proteins belonging to certain kinetic classes or functional groups. The *p*-values of Wilcoxon signed rank tests comparing expression levels of functional groups and kinetic classes (functional groups: structural, nonstructural, morphogenesis, immune evasion; kinetic classes: early, late, ambivalent) of ASFV proteins in moMΦ and WSL ranged between 0.567 and 1 (Table S3).

Despite the absence of systematic biases regarding kinetic classes or functional groups and a good correlation (R = 0.81) of the expression levels of ASFV proteins between cell types (Figure 4), ten viral proteins were expressed at significantly different levels between the cell types. Specifically, the expression of eight viral genes (H124R, H233R, H240R, G1340L, NP868R, A224L, C717R and K145R) was more abundant in WSL cells, while A151R and F334L were expressed at higher levels in moMΦ (Figure 5C).

Furthermore, comparison of the absolute protein abundances of the most abundant ASFV proteins revealed some notable differences. While some proteins ranked under the top 20 most abundant only in one cell type, for example CP123L in moMΦ or CP80R and K78R in WSL cells, others such as K145R, CP312R, A104R and C129R were abundantly expressed in both cell types (Figure 5D).

### 3.4. Response of Host Proteome to ASFV Infection

The host response of moMΦ and WSL cells to infection with ASFV was compared by monitoring the change of expression levels of individual host genes after the infection of both cell types (Figure 3, Supplementary File ‘S4_MS-statistics.xlsx’, sheet ‘Q_Effect Infection’) and by GO term and KEGG pathway enrichment analysis on the basis of lists with DEGs (Supplementary File ‘S5_Gene_lists.xlsx’, sheet ‘Gene lists for EA’). 

Since not all CD markers were present in WSL cells, the effect of ASFV infection on their expression could only be assessed in moMΦ. While the levels of most of the detected CD markers were not affected by ASFV infection, CD14 and CD74 levels significantly dropped after infection (Figure 3B).

The analysis of gene expression levels and enrichment analysis comparing infected moMΦ and WSL cells showed that similar genes were differentially expressed and GO-terms enrichments were again dominated by the characteristics of moMΦ as phagocytic, antigen-presenting cells (Supplementary File ‘S5_Gene_lists.xlsx’, sheet ‘Results enrichment analysis’).

For the majority of host genes, no significant alteration of expression levels was noted in response to ASFV infection in either cell type. Interestingly, while up- and downregulation was quite balanced in WSL cells, downregulation dominated in moMΦ (Figure S4b). Among the downregulated genes in moMΦ were heme oxygenase 1 (HMOX1), syndecan binding protein (SDCBP1), the catalytic subunit of protein phosphatase 1 (PPP1CB) and also MRC1 and SOD2, which were not detected in WSL cells (Figure 3C). Additionally, lysosomal-associated enzymes (e.g., HEXA, HEXBA, CTSA and Cathepsin H) were downregulated upon the ASFV infection of moMΦ, resulting in an under-representation of KEGG pathways related to lysosome (KEGG:04142), endocytosis (KEGG:04144) and phagosome (KEGG:04145) in ASFV-infected moMΦ in comparison to naïve moMΦ. Furthermore, moMΦ pathways related to oxidative phosphorylation (KEGG:00190) and protein processing in endoplasmic reticulum (KEGG:04141) were enriched after infection, with only oxidative phosphorylation also being enriched in infected WSL cells (Supplementary File ‘S5_Gene_lists.xlsx’, sheet ‘Results enrichment analysis’).

## 4. Discussion

In this study, we infected the natural target cell for ASFV, i.e., macrophages and the porcine cell line WSL-R, to analyze the expression levels of the viral proteins and the response that ASFV infection elicits regarding the expression of host proteins.

Taking the proteome studies of Keßler et al. [2], Alejo et al. [3] and Wang et al. [19] and other studies summarized by Dixon and colleagues [1], the number of ASFV ORFs for which protein products have been detected added up to 123 prior to this study. This still leaves a ‘dark’ ASFV proteome of approximately 44% of the ORFs for which the evidence of a corresponding protein expression is still lacking. Until now, approximately 1/3 of all described ASFV genes lacked any confirmation of expression.

Overall, we detected the expression of 112/167 ORFs (67%) present in the used genotype IX isolate, including nine members of the MGFs 100, 110, 360 or 505. We confirmed the expression of 86/94 genes previously described during ASFV-related intracellular proteome studies using the genotype I isolate OURT88/3 [2]. Of the remaining eight unconfirmed ORFs (I177L, I196L, L60L, MGF 110-14L, MGF 110-2L, MGF 110-5L and O61R), seven are present in the Kenya1033 strain, supporting the hypothesis that viral isolates of different pathogenicity (highly pathogenic vs. adapted) and genotype (genotype I vs. genotype IX) may have different requirements regarding expressed viral proteins for successful infections.

Of the 84 ORFs coding for viral proteins that were detected in the viral particle [3,19] two were not present in the used isolate (EP402R coding for CD2v [54] and ASFV_G_ACD_01760 coding for an uncharacterized protein). Three of the eight cleavage products of polyproteins pp220 (ORF CP2475L) and pp60 (ORF CP530R) were identified. The detection of the newly formed N-temini of the cleavage products p8 and p37 suggest that the maturation of pp220 and pp60 was completed, as these are thought to be generated during the final step of the polyprotein proteolytic cascade [3]. We failed to identify the gene products of MGF 360-12L, A589L and EP153R, which have been detected in viral particles [3,19], but also escaped identification in the intracellular proteome study by Keßler et al. [2], indicating that these may be expressed at low levels and require enrichment for reliable identification via MS.

In this study, we provided evidence for the existence of corresponding proteins for eleven ASFV ORFs for which previously, only transcripts had been described. For some of these, functions have been delineated from the sequence. Specifically, these are K196R, which encodes the viral thymidine kinase [44,55], B385R, a Vaccinia A2-like transcription factor [46] and E301R, a PCNA-like protein [45]. Three proteins (pD129L, pI9R and pI7L) lack any characterization so far, and five of the newly detected proteins are members of MGFs.

While transcripts of B385R and D129L genes have been described in BA71V-infected Vero cells and in the blood of Georgia 2007/1-infected animals [16,18], I9R- and I7L-specific transcripts have only been detected in blood of Georgia 2007/1-infected pigs [18]. These genes are absent in the BA71V isolate (GenBank U18466.1), which has been used for many in vitro infection experiments. Although present in genotype I strain OURT88/3 (GenBank AM712240.1) used by Keßler et al. [2] and also in the currently circulating genotype II virus descending from Georgia 2007/1 (GenBank FR682468.2) used by Wang et al. [19] (GenBank MK333180.1), I9R and I7L expression products escaped detection in both studies. This could indicate that the expression of I9R and I7L is specifically required for the replication of the used genotype IX representative, in WSL cells as well as in moMΦ.

Our main interest was to uncover differences between the ASFV gene expression profiles in the stable WSL cell line and moMΦ. Indeed, 10 viral proteins were exclusively detected in moMΦ, among them the MGF proteins pMGF 110-1L and pMGF 360-18R. The latter is a virulence factor which has been detected as a transcript in macrophages infected with the pathogenic Benin 97/1 isolate [49], but neither in a recent transcriptome study using BA71V infected Vero cells [16], nor in a proteomic study using WSL cells infected with OURT88/3 [2], indicating it may be expressed a cell-type-specific way.

It is interesting to note that in the present study, the infection of moMΦ resulted in the downregulation of a known MGF 360-16R interactor, SDCBP [47], which was not detected in WSL cells. MGF 360-16R has been described to play a role during immune evasion by inhibiting interferon signaling [47].

As observed after the infection of stable cell lines with OURT88/3 [2], the poorly characterized viral ORFs K145R, CP312R, CP123R and I73R also belonged to the most highly expressed proteins after the infection of moMΦ. CP312R is predicted to encode an EF-G-like GTPase [46], while for the others, no functional prediction has currently been made. High expression levels observed after the infection of different cell types with different virus isolates suggests an important function of these proteins for the viral life cycle. Further studies regarding their functions and interactions might help to increase the understanding of the ASFV life cycle.

While we did not observe marked differences between the expression levels of certain groups of viral genes (functional groups or kinetic classes), the expression levels of some individual viral proteins differed significantly between moMΦ and WSL cells. Higher expression levels were observed in WSL cells for pK145R, the capsid protein pH240R [19], A7L-like transcription factor G1340L [46], mRNA capping enzyme pNP868R [56] and the inhibitor of apoptosis protein pA224L [51], and the functionally uncharacterized late viral proteins pH124R, pH233R and pC717R [16]. In contrast, the ribonucleotide reductase small subunit pF334L [45] and pA151R, a known component of the virally encoded redox pathway [52], showed significantly higher expression levels in moMΦ.

These observations indicate that in general, the expression profile of ASFV proteins in primary swine macrophages does not differ from that in porcine cell cultures as WSL cells. It was, however, interesting to note that a number of so far uncharacterized viral proteins were abundantly expressed in both cells, indicating that it is possibly important to support ASFV infection in general, while the few observed differences in expression of viral proteins could be related to cellular characteristics.

Comparing the response to infection in moMΦ and WSL cells, it was striking that that a larger number of genes were downregulated in moMΦ than in WSL cells. It is known that ASFV interferes with host cell transcription [57]; however, there is no consensus as to whether ASFV induces a general host shut off [17,24,25]. Our observation could therefore be interpreted in a way that ASFV induces a host shut off in moMΦ, but not or only to a weaker extent in WSL cells. The extent of a possible ASFV-induced shut off and the possible impact on different cellular pathways could help to increase the understanding of ASFV but requires further investigation.

The morphologic and phenotypic characterization of moMΦ via flow cytometry and immune fluorescence showed characteristic features for cells of the monocyte/macrophage lineage. The phenotypic characterization of naïve moMΦ showed the expression of CD14, CD68, CD163 and SLA II on the surface of close to 100% of cells. The MS data confirmed the expression of these characteristic markers and additionally identified ubiquitously expressed CD47, CD63 (expressed by leucocytes) and CD74 (important for antigen presenting cells; reviewed in [58]). Out of these characteristic CD markers for cells of the monocyte macrophage lineage, only SLA II was detected in WSL cells. The observed changes of CD marker expression in response to ASFV infection in moMΦ is coherent with previous reports of the downregulation of CD14 [59,60], while the expression of SLA II, CD163 and CD68 appears to be unaffected by ASFV infection [59,61,62]. During ASFV infection, CD14 and CD74, described activators of NFκB-mediated signaling [63,64,65], were downregulated. NFκB-mediated signaling is targeted by ASFV immune evasion pathways by multiple mechanisms involving virally encoded proteins pA238L, pI329L or members of MGFs (reviewed in [9,66]).

For some proteins, we observed responses that are interesting to discuss in the light of published data. While the upregulation of MRC1 and HMOX1 mRNA after infection was observed previously [17,18], we noted a downregulation of MRC1 and HMOX1 protein levels in moMΦ. These observations may be of interest as HMOX1 has been described to have antiviral activities and cytoprotective effects during infection with Ebola virus [67], alpha herpes virus Pseudorabies virus [68] and SARS-CoV2 [69]. The role of MRC1 in the context of antiviral immune responses is not well characterized, but functions for antigen cross-presentation and FcR-mediated immune responses have been proposed [70].

We could not confirm the effects of ASFV infection on the expression of superoxide dismutase (SOD2) [22] and proteins related to 14-3-3-mediated signaling observed in a MALDI-PMF MS study [28]. While SOD2 was reported to be upregulated in infected Vero cells [22], we observed lower expression after the infection of moMΦ, and no alteration in WSL, indicating that the ASFV-induced regulation of SOD2 could be cell-type-specific. 

ASFV infection could have a less pronounced influence on 14-3-3-mediated signaling than previously proposed [28], since in neither cell type was an impact on 14-3-3-related GO or KEGG terms observed during the term enrichment analysis. However, this effect could also be related to the used virus genotypes (genotype IX vs. genotype I isolate E75) or the related analyzed cells (cultured cells vs. lymph nodes isolated from infected animals).

Studies investigating the early steps of ASFV infection pointed toward the importance of the endolysosomal pathway for the entry and uncoating processes of ASFV (reviewed in [71]), before viral replication can occur in cytoplasmatic viral factories. It has been proposed that endosomal components are likely to be involved in the formation of these replication sites [72]. Therefore, the observed changes in protein expression patterns in compartments of the endolysosomal pathway in moMΦ during the late stages of infection might indicate a modulation of the cellular proteome to generate conditions that allow viral replication within this specialized compartment.

In this context, the downregulation of lysosomal enzymes such as cathepsins A and H might play a role in morphogenesis. Cathepsins are a group of lysosomal proteases with important functions related to physiological processes, including cell death and immune functions. Different members of this family have been described to affect viral morphogenesis of herpes simplex virus type 1 [73], influenza A virus [74] and most recently, SARS-CoV2 [75].

Comparing ASFV infection in vitro between primary macrophages and the stable cell line WSL-R, we noted no major difference between the cell types with regard to the expression of viral genes, the regulation of host proteins or the enrichment of GO terms, besides terms related to macrophage cell characteristics. However, significant differences were found for the expression of selected viral (A151R, K145R, members of MGFs) and host genes (CD74, HMOX1 and MRC1) which deserve further investigation. Overall, our results suggest that both WSL cells and primary macrophages are suitable models for use in the study of ASFV infection in vitro, and the results of infection studies can expected to be similar. However, the observed subtle differences in the expression profiles of viral and host proteins after infection could reflect cell-specific responses to infection that may have to be considered in the design of future experiments.

## Figures and Tables

**Table 1:** Summary of viral genes that are differentially expressed in moMΦ and WSL cells or for which corresponding proteins have been identified for the first time.

ASFV Gene	Detected in		Function	References
	moMΦ	WSL		
K196R ^1^	X		Thymidine kinase	[44]
B385R ^1^	X	X	Vaccinia A2-like transcription factor ^2^	[45]
E301R ^1^	X	X	PCNA-like protein ^2^	[45]
D129L ^1^	X	X	Uncharacterized	[18]
I9R ^1^	X	X	Uncharacterized	[18]
I7L ^1^	X	X	Uncharacterized	[18]
MGF 100-2L ^1^	X	X	Uncharacterized	[18]
MGF 100-3L ^1^	X	X	Uncharacterized	[46]
MGF 110-9L ^1^	X	X	Uncharacterized	[4]
MGF 360-16R ^1^	X	X	Interactor of SERTAD3 and SFCBP	[47]
MGF 505-3R ^1^		X	IFN inhibitor ^2^	[7]
MGF 110-1L	X		Uncharacterized	[48]
MGF 360-18R	X		Uncharacterized	[49]
H124R	X	+	Uncharacterized	[2,3]
H233R	X	+	Uncharacterized	[2]
H240R	X	+	Capsid protein	[19]
G1340L	X	+	Vaccinia A7L-like transcription factor ^2^	[46]
NP868R	X	+	mRNA capping enzyme ^2^	[50]
A224L	X	+	Bcl2-homologue	[51]
C717R	X	+	Uncharacterized	[2,3]
K145R	X	+	Uncharacterized	[2,3]
A151R	+	X	Component of Redox Pathway	[52]
F334L	+	X	Ribonucleotide reductase (small SU) ^2^	[53]

^1^ confirmed expression of transcripts; ^2^ predicted function; X: detected; + overexpressed (*p* < 0.05; fold change >2).

## Data Availability

Raw data are provided within Supplementary File ‘Table S6_MS-identification.xlsx’.

## Supplementary Materials

The following files are available online at https://www.mdpi.com/article/10.3390/v13112198/s1. Figure S1: gating strategy for characterization of moMΦ by flow cytometry, Figure S2: confirmation of ASFV infection in MS samples, Figure S3: expression levels of viral proteins in individual replicates, Figure S4: volcano plots comparing expression levels of host genes under different conditions, Figure S5: detected peptides and distribution of ASFV-polyproteins ASFV-CP530R (pp62) and ASFV-CP2475L (pp220), Figure S6: peptide spectra of ASFV genes K196R and MGF 505-3R, Table S1: antibodies used for flow-cytometry, Table S2: relative standard deviations of host and viral proteins between sample groups, Table S3: results of Wilcoxon ranked test for differential expression of genes belonging to functional groups or kinetic classes in moMΦ or WSL cells, Supplementary File ‘S4_MS-statistics.xlsx’, Supplementary File ‘S5_Gene_lists.xlsx’, Supplementary File ‘S6_MS-identification.xlsx’, Supplementary File ‘S7_MS-quantification.xlsx’.
