# Supplementary material for: Comparison of the Proteomes of Porcine Macrophages and a Stable Porcine Cell Line after Infection with African Swine Fever Virus
Source: Viruses. 2021 Nov 1;13(11):2198. doi: 10.3390/v13112198 (PMC8620826; doi:10.3390/v13112198)
Supplement: Supplementary file 1 [file viruses-13-02198-s001.zip › Supplementary_information.pdf]

Supplementary Information

Title Comparison of the proteomes of porcine macrophages and a stable porcine cell line after infection with African swine fever virus

Authors Elisabeth Wöhnke, Walter Fuchs, Luise Hartmann, Ulrike Blohm, Sandra Blome, Thomas C. Mettenleiter, Axel Karger

Supplementary Methods

Characterization of moMΦ by Flow Cytometry

Table S1: Antibodies used for flow cytometry

specificity	clone	host/isotype	conjugate	source	dilution
**ZombieAqua (Cell viability)**				Biolegend	1:500
**CD14**	MIL2	mouse/IgG2b	-	Biorad	1:500
**CD172a**	BL1H7	mouse/IgG1	FITC	Biorad	1:1000
**CD68**	Y1/82A	mouse/IgG2b	PerCP-Cy5.5	Biolegend	1:20
**SLA II**	MSA3	mouse/IgG2a	-	in-house	1:100
**CD163**	2A10/11	mouse/IgG1	PE	Thermo fisher	1:100
**Mouse IgG2a**	polyclonal	goat/IgG	APC-Cy7	Southern biotech	1:250
**Mouse IgG2b**	polyclonal	goat/IgG	PE-Cy7	Southern biotech	1:400

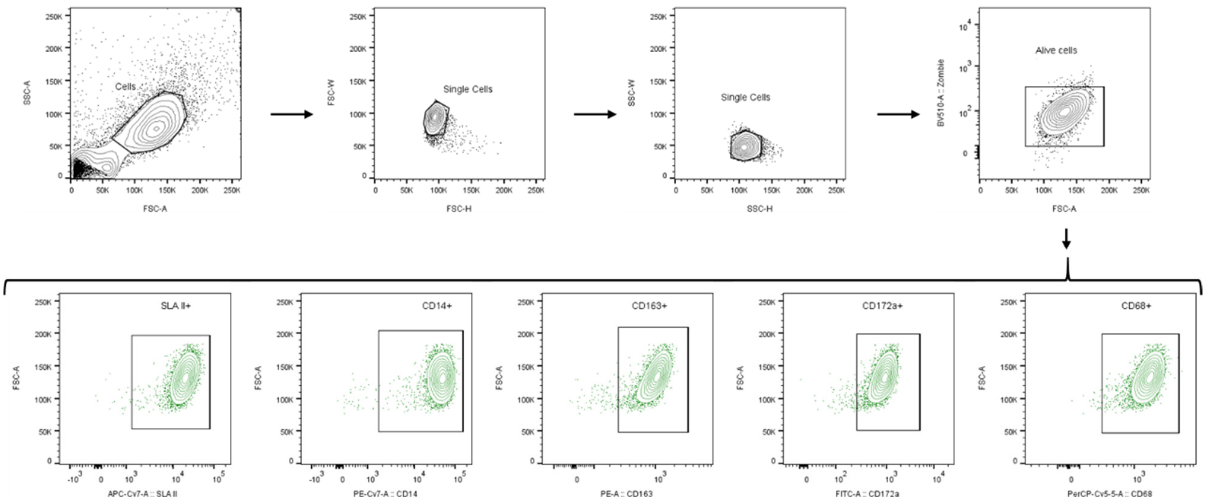

Figure S1: Gating strategy for the characterization of moMΦ by flow cytometry.

Generation and Analysis of MS-Samples

Filter Aided Sample Preparation (FASP)

Reduced cell lysates (final concentration DTT 0.5%) were digested by FASP using Trypsin (Promega #V5111) and Vivacon 500 filter units (MWCO 30 kDa, Sartorius) as previously described [4]. Digest was performed for 16h at 37°C with an enzyme-to-substrate ratio of 1:50. After filtration, peptides were desalted using Pierce™ C18 tips (ThermoScientific) as suggested by the manufacturer. Desalted peptides were dried under vacuum and dissolved in 0.1% (v/v) trifluoroacetic acid (TFA, Thermo). Peptide yields were estimated by BCA assay [5].

LC-MS/MS

Peptides were fractionated by nano reversed phase liquid chromatography (Ultimate 3000 RSLCnano, Thermo Scientific). Peptides generated from Macrophages or WSL were applied onto the pre-column (Acclaim™ PepMap™ 100, C18, 2, 3µm, 75µmx20mm, ThermoFisher Scientific), washed with 0.05% TFA (solvent A) at a flow rate of 5µl/min, and eluted onto the analytical column (Acclaim™ PepMap™ 100, C18, 2, 3µm, 75µmx15cm, ThermoFisher Scientific) applying a linear gradient of acetonitrile (ACN) from 4% to 32% solvent B (90% ACN, 0.05% TFA) with a constant flow rate of 300 nl/min. Fractions were collected every 10 seconds and automatically mixed with 0.416µL α-cyano-hydroxycinnamic acid matrix solution (prepared as suggested by the manufacturer Bruker) during spotting with a fcII spotter (Bruker).

MALDI-TOF/TOF spectra were acquired in positive mode within an m/z range of 700-3500 Da. For fragmentation, a maximum of 40 peptides per fraction (signal-to-noise ratio >5) were selected. Spectra were processed with FlexAnalysis (version 3.4, Bruker).

Proteins were identified using the Mascot search engine (version 2.7, MatrixScience, UK) [6]. Mass tolerances were set to 25ppm and 0.7 Da for peptides and fragments, respectively, and one missed cleavage site per peptide was tolerated. Methionine oxidation and acetylation of protein N-termini were allowed as options, while the carbamidomethyl of cysteine was set as fixed modification. The false discovery rate for individual runs was set to 2%. Proteins were identified using a database combining host (*S. scrofa*; downloaded from Ensembl respository [7]) and viral protein sequences.

Results of the Mascot protein searches were exported to ProteinScape (version 3.1, Bruker).

Host cell proteins considered for analysis were detected with at least two peptides. For viral proteins reported with only one unique peptide in a single run, spectra are provided in supplementary data (Figure S6).

SDS PAGE, immunoblotting and immunofluorescence analysis

Cell lysates were separated on hand casted gradient (7-15%) acrylamide gels by electrophoresis [1] and proteins were either visualized with colloidal Coomassie Brilliant Blue staining [2] or blotted to nitrocellulose membrane [3]. Viral proteins were detected on blots using rabbit sera against p30 and p72 (provided by Dr. W. Fuchs) and a peroxidase-conjugated anti-rabbit antibody. Blots were developed using Clarity Western ECL substrate (BioRad).

Supplemental Data

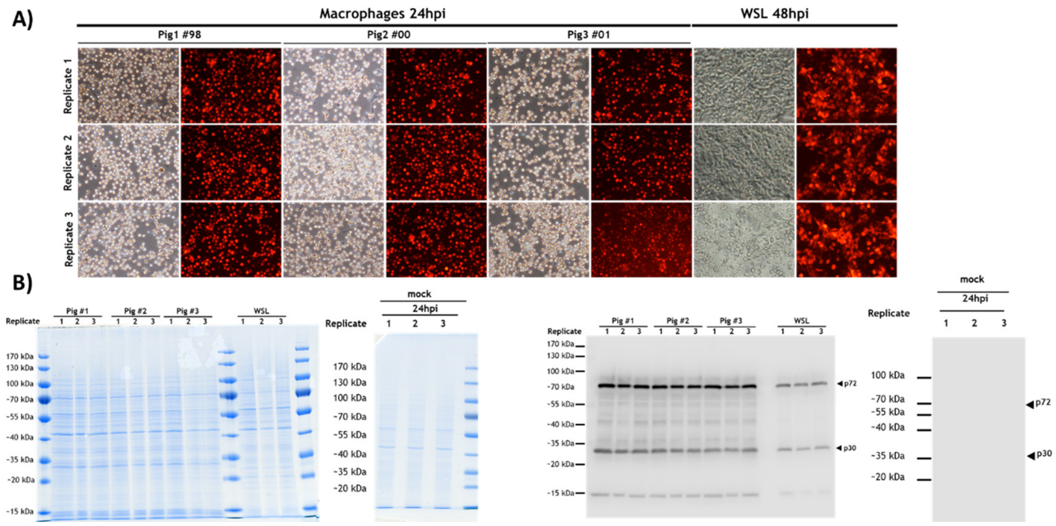

Figure S2: Confirmation of ASFV infection in cells prepared for MS analysis. (a) Detection of reporter gene dsRed, (b) detection of p30 (early) and p72 (late) viral proteins in moMΦ and WSL in immunoblots (right) together with the corresponding Coomassie-stained SDS-PAGE gels (left).

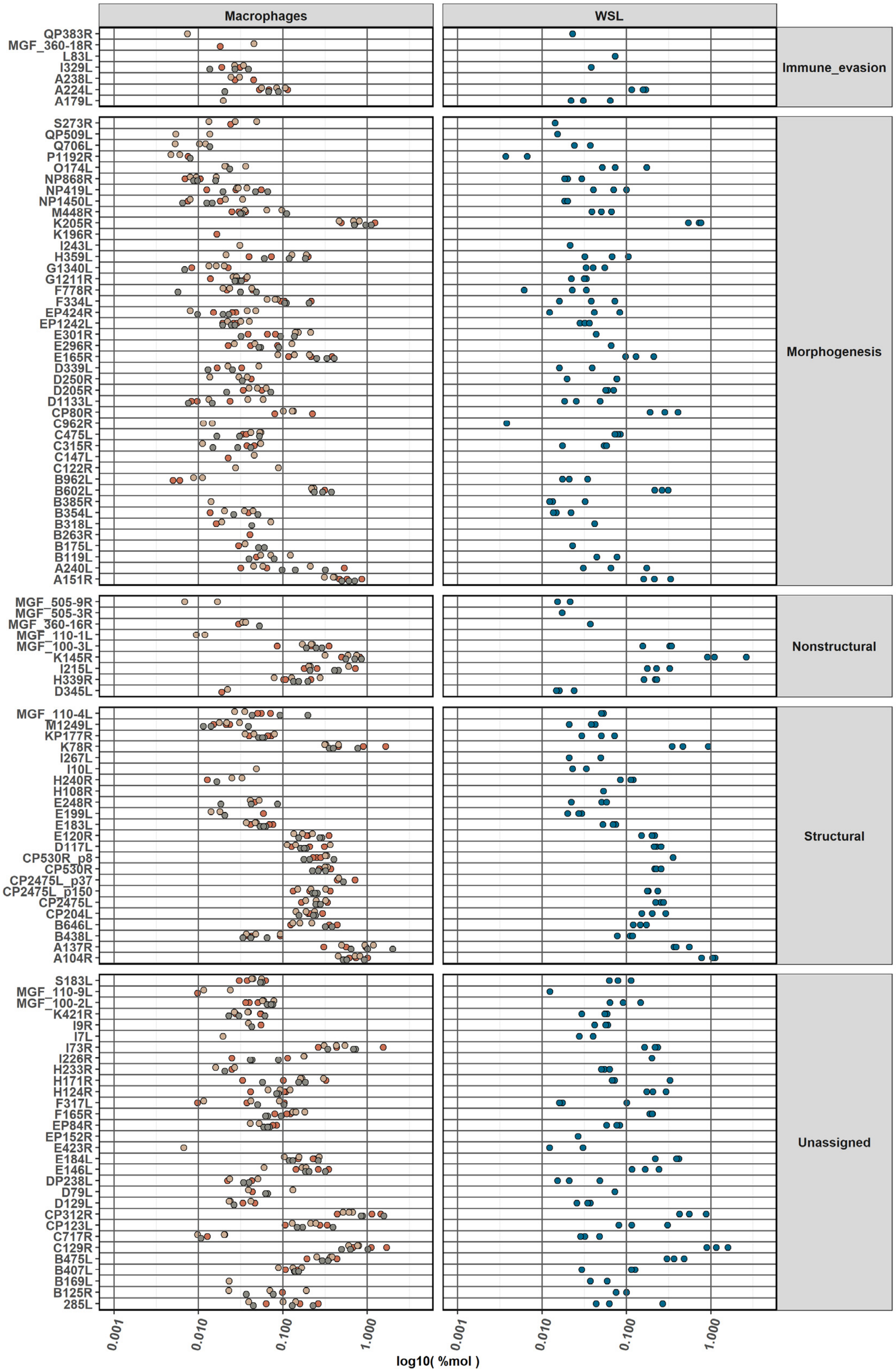

Figure S3: Expression levels of viral proteins in individual replicates. Different colors in panel 'Macrophages' represent the three pigs from which moMΦ were isolated.

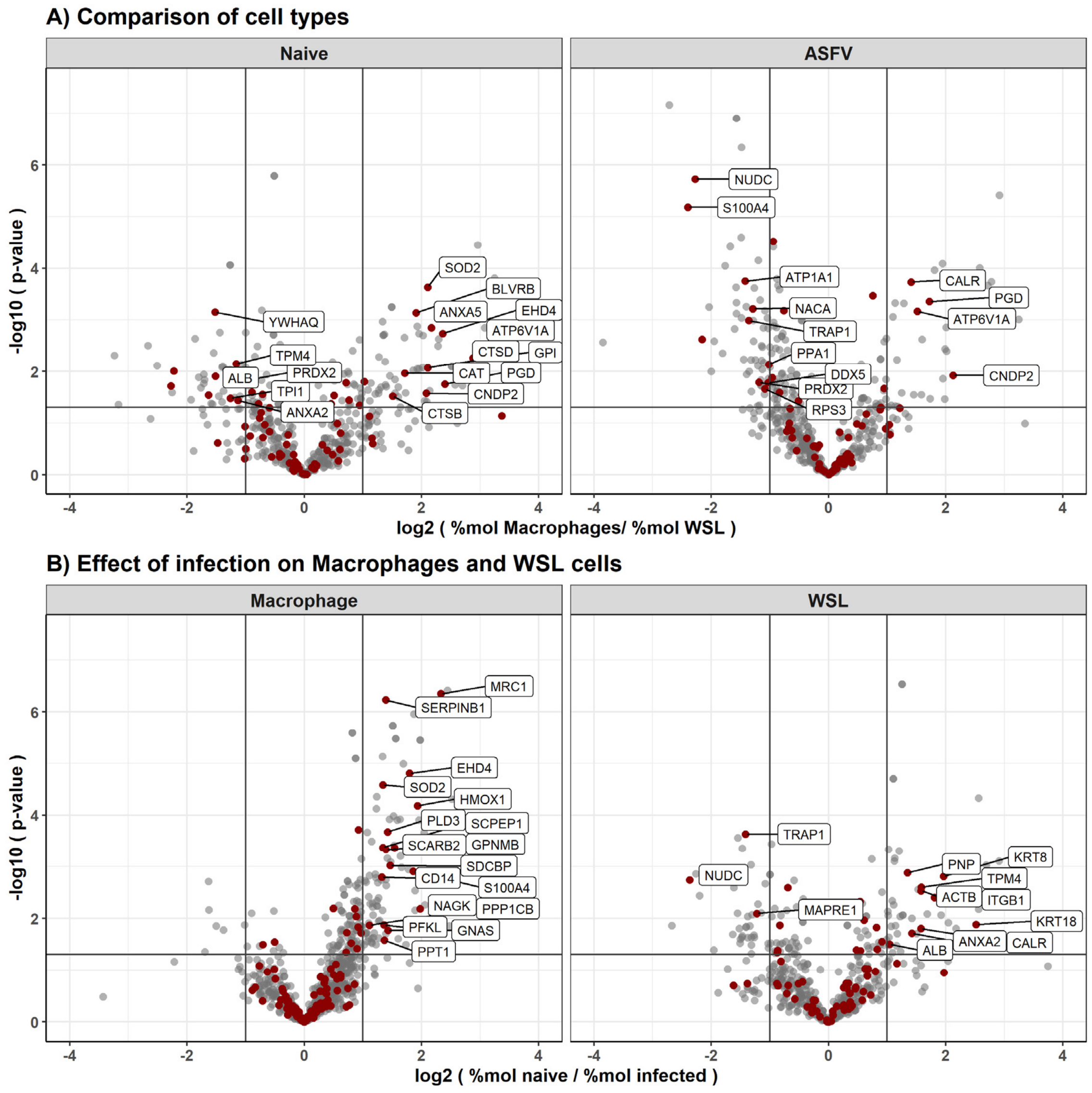

Figure S4: Volcano plots comparing the expression levels of host genes under different conditions. (a) Comparison of moMΦ and WSL cells in naïve (left) and infected (right) cells. (b) Comparison of naïve to infected moMΦ (left) and WSL (right). Red dots mark genes previously highlighted in publications related to ASFV

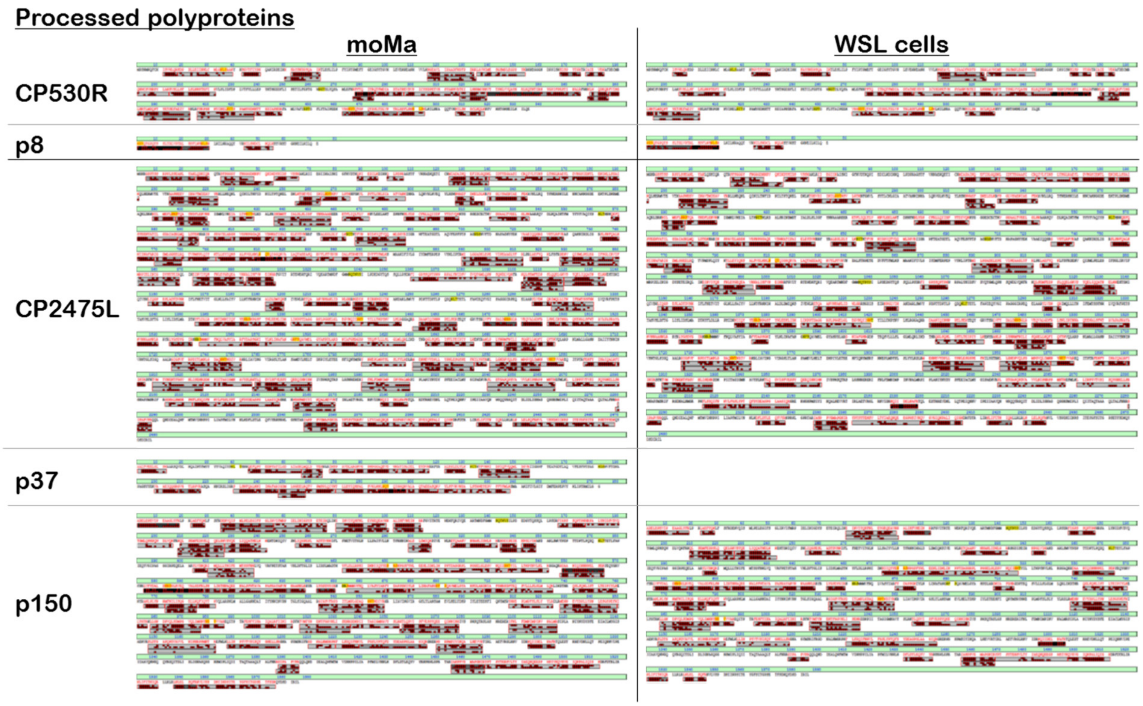

Figure S5: Peptides detected in proof of ASFV-polyprotein processing ASFV-CP530R (pp62) and ASFV-CP2475L (pp220). Bars under the sequences indicate the positions of identified peptides, red boxes inside bars indicate amino acids identified by tandem MS analysis.

K196R

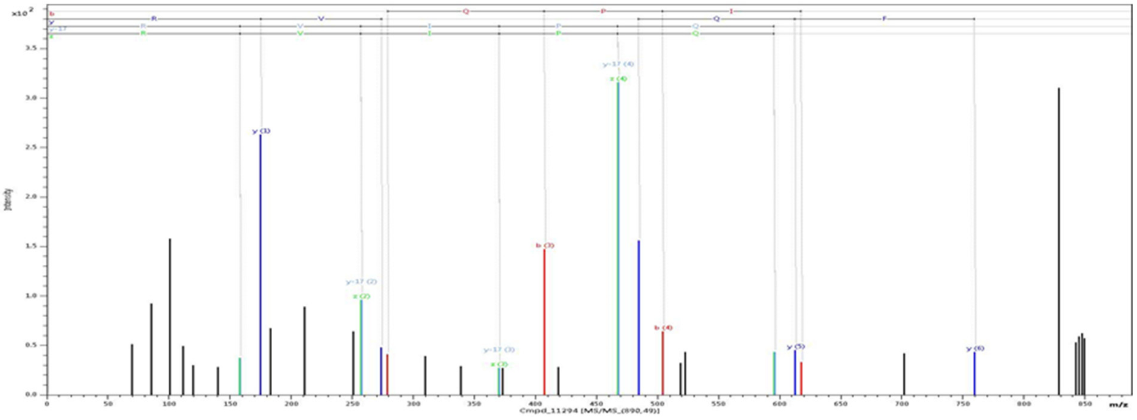

MGF 505-3R

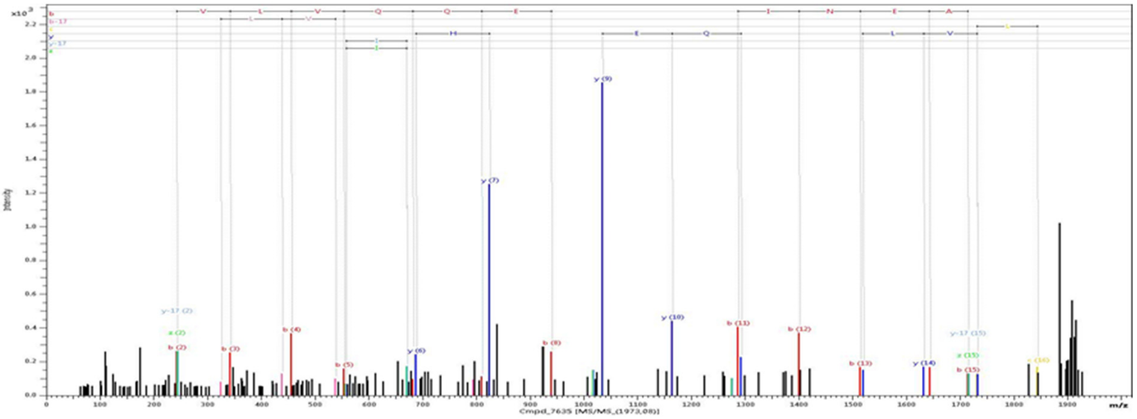

Figure S6: Peptide spectra in proof of expression of ASFV genes K196R and MGF 505-3R identified by a single unique peptide. Lines in the upper part of the graph indicate fragmentation series with identified amino acids annotated.

Table S2: Relative standard deviations of host and viral proteins between sample groups

**Celltype**	**Macrophage**		**Pig1**	**Pig2**	**Pig3**	**WSL**	
**sample**	**infected**	**mock**	**infected**	**infected**	**infected**	**infected**	**mock**
**host**	0.356	0.304	0.336	0.355	0.322	0.306	0.281
**virus**	0.411		0.356	0.454	0.373	0.352	

Table S3: Results of Wilcoxon ranked test for differential expression of genes belonging to functional groups or kinetic classes in moMΦ or WSL cells.

**Class**	**Group**	**p-value**
**Functional**	Structural	0.863
	Morphogenesis	0.762
	Nonstructural	1
	Immune_evasion	0.667
	Unassigned	0.583
**Kinetic**	early	0.758
	late	0.568
	ambivalent	0.757
	unassigned	0.69

Table S4: (file 'S4_MS-statistics.xlsx') Quantitative MS-Results for host and viral genes and results of statistical test for enrichment of genes

Table S5: (file 'S5_Gene_lists.xlsx') Gene lists used for GO and KEGG-analysis and results of CytoScape-ClueGO analysis

Table S6: (file 'S6_MS-identification.xlsx') Results of protein identifications using Mascot search engine exported from ProteinScape.

References

1. Laemmli, U.K. Cleavage of structural proteins during the assembly of the head of bacteriophage T4. *Nature* **1970**, *227*, 680–685, doi:10.1038/227680a0.
2. Neuhoff, V.; Arold, N.; Taube, D.; Ehrhardt, W. Improved staining of proteins in polyacrylamide gels including isoelectric focusing gels with clear background at nanogram sensitivity using Coomassie Brilliant Blue G-250 and R-250. *Electrophoresis* **1988**, *9*, 255–262, doi:10.1002/elps.1150090603.
3. Towbin, H.; Staehelin, T.; Gordon, J. Electrophoretic transfer of proteins from polyacrylamide gels to nitrocellulose sheets: procedure and some applications. *Proc Natl Acad Sci USA* **1979**, *76*, 4350–4354, doi:10.1073/pnas.76.9.4350.
4. Wiśniewski, J.R.; Zougman, A.; Nagaraj, N.; Mann, M. Universal sample preparation method for proteome analysis. *Nature Methods* **2009**, *6*, 359–362, doi:10.1038/nmeth.1322.
5. Stoscheck, C.M. [6] Quantitation of protein. *Guide to Protein Purification*; Elsevier, 1990; pp 50–68, ISBN 9780121820831.
6. Perkins, D.N.; Pappin, D.J.C.; Creasy, D.M.; Cottrell, J.S. Probability-based protein identification by searching sequence databases using mass spectrometry data. *Electrophoresis* **1999**, *20*, 3551–3567, doi:10.1002/(SICI)1522-2683(19991201)20:18<3551:AID-ELPS3551>3.0.CO;2-2.

7. Aken, B.L.; Achuthan, P.; Akanni, W.; Amode, M.R.; Bernsdorff, F.; Bhai, J.; Billis, K.; Carvalho-Silva, D.; Cummins, C.; Clapham, P.; et al. Ensembl 2017. *Nucleic Acids Research* **2017**, *45*, D635-D642, doi:10.1093/nar/gkw1104.
